# Deciphering the Origins of Commercial Sweetpotato Genotypes Using International Genebank Data

**DOI:** 10.3390/biology15010091

**Published:** 2026-01-01

**Authors:** Alexandre F. S. Mello, Ronald Robles, Genoveva R. M. de Simon, Giovani O. da Silva, Sonia M. N. M. Montes, Maria U. C. Nunes, Jose L. Pereira, Erich Y. T. Nakasu, Rainer Vollmer, David Ellis, Verónica Valencia-Límaco, Vânia C. R. Azevedo

**Affiliations:** 1 Embrapa Vegetables, Brasília 70351-970, DF, Brazil; giovani.olegario@embrapa.br (G.O.d.S.); jose.luiz@embrapa.br (J.L.P.); erich.nakasu@embrapa.br (E.Y.T.N.); 2 International Potato Center, Lima 1558, Peru; r.robles@cgiar.org (R.R.); g.rossel@cgiar.org (G.R.M.d.S.); r.vollmer@cgiar.org (R.V.); d.ellis@cgiar.org (D.E.); veronica.valencia@cgiar.org (V.V.-L.); v.azevedo@cgiar.org (V.C.R.A.); 3 Agência Paulista de Tecnologia dos Agronegócios, APTA, Presidente Prudente 19015-970, SP, Brazil; sonimontes1@gmail.com; 4 Embrapa Coastal Tablelands, Aracaju 49025-040, SE, Brazil; maria-urbana.nunes@embrapa.br

**Keywords:** *Ipomoea batatas* (L.) Lam., SSR markers, morphological descriptors, landrace and cultivar identification

## Abstract

Sweetpotato genotypes, often known by regional names, are easily propagated via cuttings, which can lead to mixing and misidentification of cultivars. This complicates traceability and commercialization. Accurate characterization of common genotypes would support their formal registration and strengthen the sweetpotato value chain. Sweetpotato is a staple crop in Brazil, and in this study, four states, representing different geographic regions in Brazil, were selected. A total of 37 samples were collected in these states, and the samples were evaluated by SSR molecular markers and morphological traits. The samples were cleaned of virus and compared to the global sweetpotato collection held at the International Potato Center under the International Treaty on Plant Genetic Resources for Food and Agriculture. SSR markers effectively distinguished among accessions. The genotype locally known as “Canadense” matched closely both genetically and morphologically to the CIP accession ‘Blesbok’. This alignment paves the way for formalizing cuttings and root production of “Canadense”/‘Blesbok’ for commercial use. In contrast, several accessions marketed in Sergipe as “white skin sweetpotato” did not correspond to any known CIP accession, suggesting that they may be unique regional genotypes or acquired from other sources, since sweetpotato is an exotic crop in Brazil. Overall, the research identified key genotypes, supporting their official registration with Brazil’s Ministry of Agriculture, Livestock, and Supply, thereby enhancing the legal commercialization of cuttings and roots. Additionally, the clear molecular and trait-based classification will assist sweetpotato crop improvement programs in selecting appropriate parent lines for future crosses.

## Simple Summary

In Brazil, many sweetpotato varieties are grown under local names, making it hard to know exactly what type they are. Since sweetpotatoes are easy to grow from cuttings, different cultivars often become mixed up. This causes problems with selling them legally and tracking their origin. To help fix this, researchers studied 37 sweetpotato samples from four Brazilian states. They used DNA tools (called SSR markers) and looked at physical traits to compare them with sweetpotato varieties kept at the International Potato Center (CIP). The results showed that a popular variety known as “Canadense” in Brazil matched with less than a 0.05 Jaccard dissimilarity with the cultivar ‘Blesbok’. This finding can help growers and sellers officially register and market their sweetpotato crops. Another group of sweetpotatoes, called “white skin sweetpotato” from Sergipe, did not match any known accessions in the CIP collection. Overall, the study helps identify key sweetpotato types in Brazil, which will support their official registration with MAPA (the Ministry of Agriculture) and improve how sweetpotatoes are grown, sold, and used in breeding programs.

## 1. Introduction

Sweetpotato (*Ipomoea batatas* (L.) Lam.) belongs to the Convolvulaceae family and is native to the Americas [1]. It has high nutritional value, with complex carbohydrates and a moderate glycemic index; depending on the genotype, sweetpotato can also be a good source of vitamins, minerals, and antioxidants [2,3]. In addition to its nutritional importance, the crop’s great agronomical resilience makes it a staple in several regions around the world [4].

As sweetpotato is a clonal crop, cuttings are the most common propagative material used in commercial planting [5]. This characteristic increases the likelihood of homogeneity in the crop and allows for the prompt distribution of genotypes among farmers. Despite these advantages, the long-term multiplication of clonal crops under field conditions can lead to the accumulation of pests and pathogens, particularly viruses, which can reduce crop yield and obscure genotype identity [6]. Additionally, the ease of propagation can also result in genotype mixing, leading to a misidentification of the cultivar being planted.

Sweetpotato is cultivated in most Brazilian municipalities [7]. The size of the planting areas and the degree of technology adopted vary greatly across different planting regions. However, one common aspect is that most of the sweetpotato genotypes commercially planted are not registered as commercial cultivars with the Brazilian Ministry of Agriculture, Livestock and Food Supply (MAPA) [8]. This lack of registration makes the traceability process unfeasible, directly impacting commercialization.

To address this issue, we characterized the prevalent genotypes planted in key regions of Brazil using molecular and morphological techniques in a study conducted in partnership between the Brazilian Agricultural Research Corporation (Embrapa) and the International Potato Center (CIP). CIP hosts the largest sweetpotato Genebank in the world, held in trust for humanity under the FAO International Treaty on Plant Genetic Resources for Food and Agriculture (ITPGRFA), which includes genotypes from several countries, including more than 100 samples from Brazil [9]. The genebank has its entire collection already genotyped, allowing other institutions to compare their genotypes with those hosted by CIP [10]. This molecular identification, along with the passport data held by the genebank [11], may enable the identification or at least suggest the origin of a genotype. Beyond variety identification, combining molecular and morphological tools can facilitate the detection of potential duplicate accessions, thereby supporting the development of a more streamlined collection.

In this context, the aim of this study was to assess the origin and diversity of the most planted sweetpotato genotypes in four different states in Brazil. The findings of this study directly led to the identification of several relevant genotypes that will support the registration of these varieties with MAPA, thus improving the formalization of the sweetpotato commercial chain in Brazil. Additionally, the current findings provide insight into the potential new genetic pools not yet preserved at CIP that should be sent to this international collection for proper back-up.

## 2. Materials and Methods

### 2.1. Plant Material

**Figure 1:**
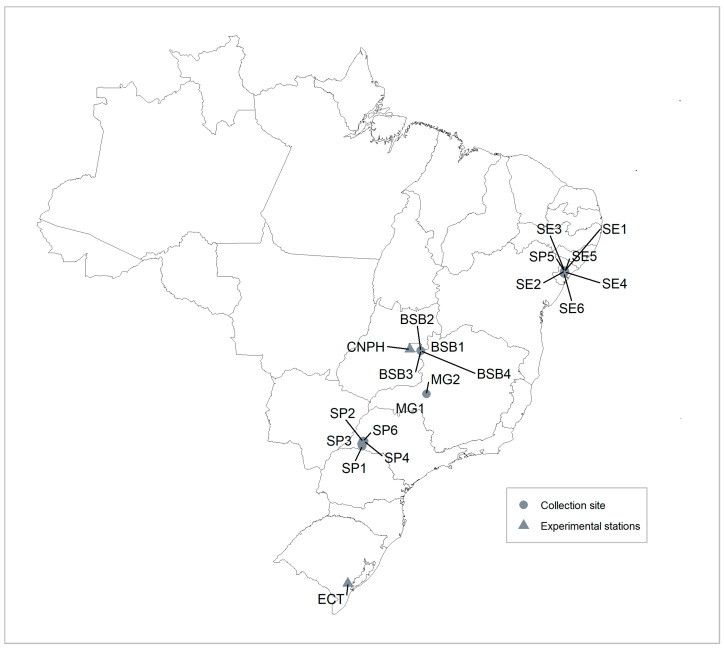
Commercial field collecting sites and experimental station locations where samples utilized in the current study were obtained. SP: São Paulo, MG: Minas Gerais, BSB: Brasília, SE: Sergipe, CNPH: Embrapa Vegetables, ECT: Embrapa Temperate Agriculture.

A total of 37 plant samples were evaluated in this study, 26 of which were collected from commercial sweetpotato growers across four different federal units of Brazil (Distrito Federal, São Paulo, Minas Gerais, and Sergipe). Five cuttings from each potential variety were collected at each location. After the in vivo establishment under greenhouse conditions, only one single plant per variety and location was selected, maintained, and used for DNA extraction, virus cleaning procedures, and morphological evaluations. The remaining plants were eliminated. Additionally, 11 cultivars were obtained from two Embrapa Research Units (Table 1, Figure 1). To minimize any impact on morphological evaluations, plants were virus-cleaned using meristem extraction from single meristems and virus indexed using NCM-ELISA for ten different viruses: sweet potato feathery mottle virus (SPFMV), sweet potato chlorotic stunt virus (SPCSV), sweet potato mild mottle virus (SPMMV), sweet potato latent virus (SPLV), sweet potato chlorotic flecks virus (SPCFV), sweet potato mild speckling virus (SPMSV), sweet potato C6 virus (SPC6V), sweet potato collusive virus (SPCV), sweet potato virus G (SPVG), and cucumber mosaic virus (CMV) [12]. Stems of the harvested samples were planted in 5 L plastic pots with commercial substrate (vermiculite) and kept under greenhouse conditions until the evaluations were completed.

### 2.2. DNA Isolation

Young fresh leaves from each individual plant sample from the greenhouse-maintained plants were collected, and DNA was extracted using the CTAB method previously described [13]. DNA quality was assessed using a Thermo Scientific NanoDrop 2000 Spectrophotometer, (Wilmington, DE, USA) while quantity was estimated with a 1% agarose gel using lambda DNA as a standard. The DNA was then diluted to 20 ng/μL for Polymerase Chain Reaction (PCR) amplification and shipped to CIP in Lima, Peru, for SSR analysis.

### 2.3. SSR Analysis

Nineteen SSR loci were selected previously from a previous study of sweetpotato characterization [10], based on empirical evaluation of their technical performance and informativeness. The selection prioritized markers showing clear and reproducible amplification patterns, high polymorphism, good allele size resolution, and low frequency of stutter bands or ambiguous profiles. These criteria ensured the use of reliable and highly discriminative loci suitable for genotyping in sweetpotato.

All forward primers were tailed with the universal M13 sequence, labeled with a fluorescent dye for detection of PCR products on a polyacrylamide gel, which were analyzed using a LI-COR IR2 4300 Global DNA analyzer (LI-COR, Lincoln, NE, USA).

The total reaction volume for each individual PCR was 10 μL. The amounts of each component and the primer combinations for multiplexing were previously reported [10]. Thermocycling conditions consisted of one cycle at 94 °C for 4 min, followed by 31 cycles of 94 °C for 1 min, 60–62 °C for 1 min (depending on the primers used), and 72 °C for 1 min, followed by an extension cycle at 72 °C for 10 min, and then held at 4 °C. All PCRs were performed in a Veriti^TM^ R 96-Well Thermal Cycler from Applied Biosystems (Waltham, MA, USA).

The PCR products were combined with 5 μL of Blue Stop Solution (LI-COR, Inc., Lincoln, NE, USA) containing bromophenol blue and formamide. The final mix was denatured at 95 °C for 3 min and immediately chilled on ice prior to gel loading on 5.5% polyacrylamide gels (KBPlus Gel Matrix, LI-COR) attached to a LI-COR IR2 4300 Global DNA Analyzer dual dye system (LI-COR, Lincoln, NE, USA). Approximately 0.2 μL from each PCR product mix was loaded into each gel lane. A 50–350 bp size standard (LI-COR, Lincoln, NE, USA) was loaded into the gel at scattered positions. Gel images were visualized with the SAGA-GT software version 3.2 (LI-COR, Lincoln, NE, USA).

### 2.4. Data Analysis

#### 2.4.1. SSR Scoring and Combined Analysis

Since sweetpotato is a polyploid species, although SSRs are co-dominant markers, alleles were scored in a binary format, “1” or “0” for the presence or absence of alleles, respectively. Missing data were coded as “9”. A preliminary analysis of the entire CIP collection (5979 accessions) and the 37 samples submitted by Embrapa was carried out by computing a Jaccard distance matrix.

The dataset of 5979 accessions corresponds to the complete SSR database of the sweetpotato collection maintained at the International Potato Center (CIP), previously reported and made publicly available as supplementary data in [10]. This reference dataset was used as a baseline to identify accessions with closely related genotype profiles to those newly analyzed in this study. By integrating the 37 samples provided by Embrapa into the global CIP dataset, we conducted a comparative clustering analysis to detect potential matches between both collections. This preliminary step ensured that the subsequent analyses focused only on relevant accessions, that is, those exhibiting close genetic relationships with the new samples.

Clustering was performed using the unweighted Neighbor-Joining algorithm implemented in DARwin software v6.0.14 [14], and the resulting dendrogram was examined to identify potential matches between both collections

A second tree was created, retaining only the accessions from this study and the matching accessions from CIP’s collection. The resulting 65 samples (37 from the current study and 28 from CIP) constitute the total number of samples in which marker statistics, diversity indexes, and dissimilarities were calculated.

#### 2.4.2. Allelic Frequencies, Polymorphism, and Diversity Estimates

Allele size ranges, number of alleles per locus, and mean number of alleles per individual were calculated for each SSR loci across all samples. Allelic frequencies were calculated using two methods. The first method considers that each allele observed in an ambiguous genotype (as in codominant markers on polyploid species) is equally likely to occur in more than one copy within the genotype. This method is implemented in SPAGEDI v1.42 and in the R package polysat (R version 4.4.0), under the function SimpleFreq.

Since this approach tends to underestimate the frequencies of common alleles while overestimating those of rare alleles, an alternative method was used. This second method, based on an EM-type algorithm for estimating allele frequencies of polyploids under polysomic inheritance [15], is implemented through the deSilvaFreq function in the R package polysat (R version 4.4.0). The selfing rate, a parameter required for the EM-type allele frequency estimation, was obtained with SPAGeDi v1.42 [16], which estimates selfing based on standardized identity disequilibrium. Because the dataset consisted of codominant SSR markers from a polyploid species without allele dosage information, the selfing rate estimated from phenotypic data was used. This value was then incorporated into the frequency estimation using the deSilvaFreq implementation [15].

Genetic diversity measurements (Nei’s gene diversity, Polymorphic Index Content (PIC)) provide valuable insights into the genetic variation and informativeness of SSR markers in the studied population, which is critical for downstream analyses like marker-assisted selection or population structure assessment. The EM-type algorithm was chosen for its ability to estimate more reliable allele frequencies in polyploid species, which often exhibit complex inheritance patterns.

Nei’s gene diversity, corrected for sample size [17], was calculated using SPAGEDI v1.42 software [16] with the formula:

$$H=\;\frac{n}{n-1}\;\left(1-\;\sum_{i=1}^{k}p_{i}^{2}\right)$$

The Polymorphism Information Content (PIC), a measure of marker informativeness, was calculated using the R package polysat [18] according to the formula from [19]:

$$PIC=1-\;\sum_{i=1}^{n}p_{i}^{2}-\;\sum_{i=1}^{n}\sum_{j=i+1}^{n}2p_{i}^{2}p_{j}^{2}$$

#### 2.4.3. Discriminatory Power Index 

To compare the capacity of the SSR locus to differentiate between samples, the Discrimination Power ${(D}_{j})$ was calculated using a formula previously reported [20]:

$$D_{j}=1-C_{j}=1-\;\sum_{i=1}^{I}p_{i}\;\frac{\left(N\;p_{i}-1\right)}{N-1}$$

where $p_{i}$ is the frequency of the ith pattern (*i* = 1, 2, 3…, *I*) observed for a given jth locus, I is the total number of patterns observed at that locus, and N is the total number of samples. The discriminatory power ${(D}_{j})$ is the complementary probability of the confusion probability ${(C}_{j})$, which is defined as the probability that two randomly chosen samples share the same banding pattern.

To test if the total number of loci is sufficient to achieve the maximum differentiation in the set of samples, a maximization curve was obtained by sequentially adding the loci that provide the highest combined Discrimination Power value. The response of the Discrimination Power curve, as the number of loci increases, indicates how many loci are necessary to achieve the same level of discrimination as when all loci are analyzed together.

#### 2.4.4. Clustering and Identification of Duplicates

A genetic dissimilarity matrix among accessions was calculated using the Jaccard dissimilarity coefficient in DARwin software v6.0.14, based on the previous binary matrix of SSR allele presence/absence treated as independent dominant markers. To assess the robustness of the tree topology, 1000 bootstrap replications were performed within DARwin distance computation.

An unweighted Neighbor-Joining algorithm was used to construct an unrooted genetic distance tree, with default parameters. The resulting tree was exported in Newick format and annotated using the Interactive Tree of Life (iTOL v7.2) [21].

Putative duplicates were identified based on the pairwise dissimilarity. Accessions previously classified as duplicates in the CIP collection show dissimilarity values of less than 0.05. Here, this threshold was used to flag candidate duplicates. Sample pairs falling below this threshold were manually inspected in the NJ tree and verified across each SSR loci to confirm matching allele profiles.

### 2.5. Morphological Evaluations

To confirm the findings observed in the molecular evaluation, a field trial was conducted at the Embrapa Vegetables station in Brasília, DF, Brazil. The 37 accessions were planted in randomized blocks, with two repetitions, where each block contained all the accessions that were closely related according to the molecular evaluation. Each experimental plot included 10 plants per accession, with two replications and 0.25 m spacing between plants. To avoid foliage and root mixing during evaluation and harvesting, plots were spaced 3.4 m along the row, with three rows on each side of the planted row (2.55 m from each side). Planting took place on May 2, 2022, and harvesting on 9 November 2022. Pre-planting fertilization included P_2_O_5_ (196.8 kg ha^−1^), Ca (76.8 kg ha^−1^), and K_2_O (58 kg ha^−1^), using triple superphosphate and KCl. Weed control was carried out 30 and 60 days after planting, followed by the application of 30 kg ha^−1^ of N as calcium nitrate.

Plant and foliage characterization was performed 90 days after planting. Evaluations included plant type, leaf size, petiole length, vine diameter and length, immature and mature foliage color, leaf general outline, lobe type and number, central lobe type, leaf vein pigmentation, vine pubescence and pigmentation [22]. Plants were harvested 191 days after planting, and roots were characterized according to shape, defects, primary and secondary skin and flesh colors, distribution of secondary flesh color, and cortex thickness [22]. Data was compared with the passport information of CIP accessions to evaluate similarity.

## 3. Results

### 3.1. Polymorphism and Diversity Estimates

The nineteen SSR loci produced a total of 156 clear and scoreable alleles, which were registered in a binary format (1/0) for the presence or absence of alleles. The number of alleles per locus ranged from 3 to 12 (for IBS14 and IBS139, respectively), with an average of 8.21 scored alleles per locus. Previous studies using SSR markers in sweetpotato have reported comparable or slightly higher allelic diversity. A previous study analyzed 130 accessions with 13 SSR loci (including IbJ522A, IbJ10a, IbJ116A, IBS11, and IbJ544b) and found a mean allele number of 10.38 [23], while [1] evaluated 139 accessions with eight SSR loci (four of which were also used in this study: IbJ544b, IbJ116A, IBS11, and IbJ522A) and reported a mean allele number of 12.38, attributed to the broader and more diverse sample set selected to detect population structure signals.

At the individual level, there was a maximum of six different alleles in a single individual, and the mean number of alleles per individual ranged from 2.08 to 4.42 (for loci IBS14 and IBS28, respectively) (Table 2).

Nei’s measure of genetic diversity, when corrected for sample size, ranged from (0.629) to (0.873), with an average value of 0.80. Similar results were obtained when the correction was not applied. The greatest variation after correction was observed for IbJ544b, with a value ranging from 0.6254 to 0.6290, representing a 0.58% variation. The PIC, when calculated using the SimpleFreq method, produced values ranging from 0.56 to 0.86, with an average of 0.77 (Table 2). Using the EM-type estimation, implemented in deSilvaFreq with an obtained selfing rate value of 0.07 to derive corrected allelic frequencies according to [15], PIC values ranged from 0.55 to 0.84, with an average value of 0.75. More variation was observed between the PIC estimates calculated with and without correction of allele frequencies, with the greatest variation occurring in IBS14, where PIC values changed from 0.57 to 0.64, representing a 13.37% variation. In all cases, the loci with the minimum and maximum PIC values were IbJ544b and IBS141, respectively. The complete list of values is shown in Table 2.

Nei’s gene diversity and the PIC are key indicators of the level of genetic variability within a set of accessions. It is important to note that the samples analyzed here were not selected to maximize genetic diversity but rather consist of accessions with very close genetic distances, as the main objective of this study was to identify potential duplicates. Despite this, both parameters indicate that the SSR loci successfully captured allelic variation among sweetpotato samples, allowing clear differentiation among genotypes.

**Figure 2:**
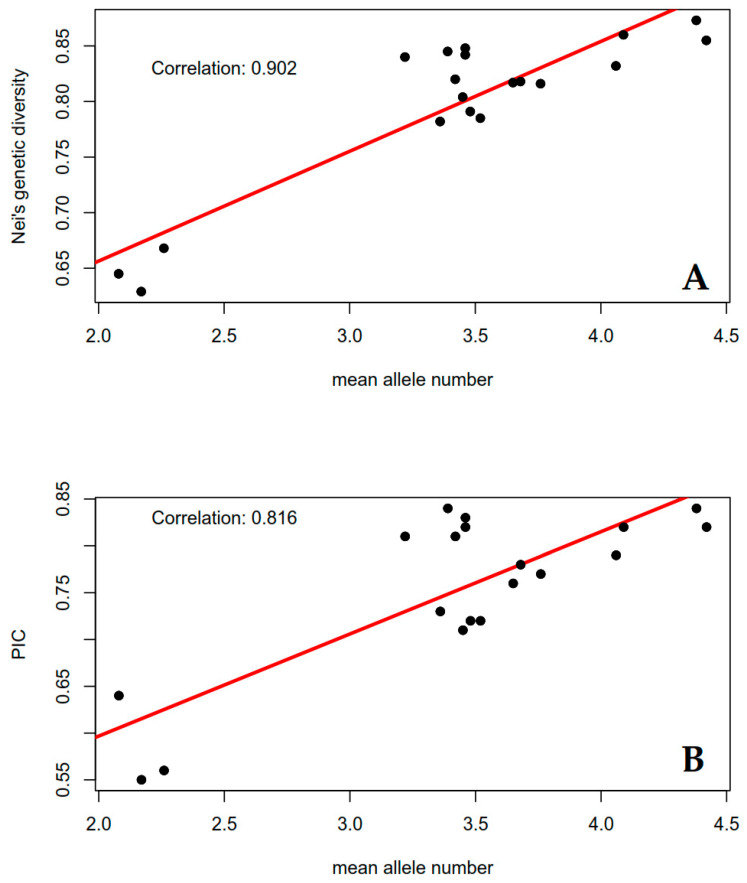
Correlation plots showing the relationships between (**A**) allele number and Nei’s genetic diversity, and (**B**) mean allele number and Polymorphism Information Content (PIC). The strong positive correlations (r = 0.902 and r = 0.816, respectively) indicate that loci with a higher number of detected alleles tend to display greater informativeness and polymorphism, confirming that allele richness is a major contributor to the discriminatory power of the SSR markers used in this study.

The Nei’s gene diversity and PIC estimates increase, as does the mean number of alleles; this was verified with strong positive Pearson’s correlation values of 0.902 and 0.816, respectively (Figure 2). We observed that loci with the lowest number of alleles exhibited more variation when allele frequencies were corrected to calculate the PIC estimates. This is consistent with the greater impact of the correction when fewer alleles are involved in the algorithm, whereas in loci with more alleles, the effect of the correction is diluted across the allele frequencies.

### 3.2. Discrimination of Samples

The discrimination power of the loci ranged from 0.72 to 0.93 (for loci IbJ544b and IBS28, respectively) with an average of 0.88. These estimates of discrimination power (*D_j_*) are influenced by the level of redundancy in the current set of samples; hence, they are useful only for comparing the differentiation ability within this dataset. Fourteen of the SSR loci distinguished more than 15 different genotype band patterns, with a maximum of 22 for the loci IBS28 and IBS139. In contrast, four SSR loci exhibited poor discrimination power, detecting only six (IBS14) and eight (IbJ522a and IbJ544b) different banding patterns (Table 3).

Assuming the non-correlation of loci, the discriminatory capacity of the combined use of all SSR loci would be equal to the product of their individual values. However, this is not the case, as not all markers behave truly independently. To address this issue, the combined Discriminatory Power (D_j_) was calculated by sequentially increasing the number of loci used, thus estimating the minimum set of SSR loci required to achieve maximum discrimination.

**Figure 3:**
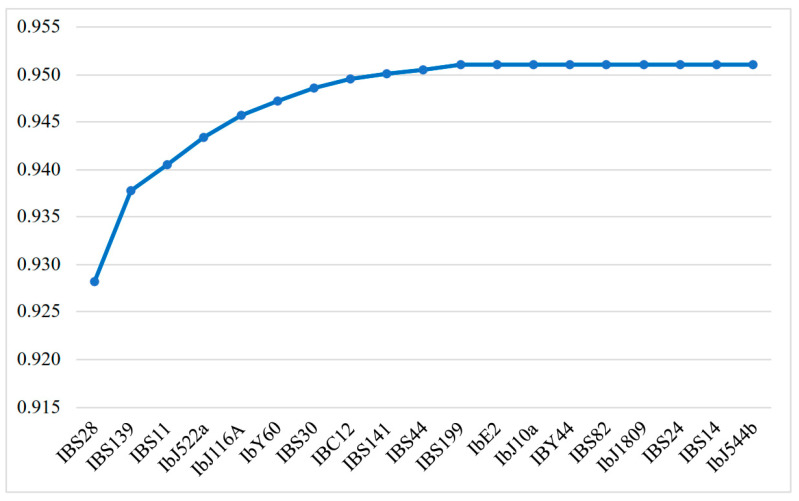
Cumulative increase in discriminatory power obtained by the successive addition of SSR loci in order of their contribution to genotype differentiation. The curve reaches a plateau at 0.951 after the inclusion of eleven loci, indicating that the entire set was sufficient to maximize individual discrimination across all samples.

The procedure began with the locus showing the highest individual discriminatory power, followed by the sequential inclusion of additional loci that, in combination with those already selected, produced the next highest cumulative value of discriminatory power. At each step, all remaining loci were tested, and the one that maximized the overall discrimination among genotypes was retained. The discriminatory power reached its maximum value (detecting 37 different banding patterns) when 11 SSR loci were used. These 11 loci achieved an accumulated discriminatory power of 0.951, whereas the addition of the remaining SSR loci provided additional resolution of the relationships between accessions (Figure 3). This implies that the nineteen SSR loci used in this study were sufficient to detect more than 95% of the possible variations between accessions (Figure 3). Interestingly, the 11 selected SSR loci exhibited the highest values in diversity estimates (Nei diversity, heterozygosity, and PIC), as shown in Table 2. This characteristic correlates with their high number of alleles per locus.

### 3.3. Comparison of Samples Collected in This Study and the Ones Present at CIP Collection

**Figure 4:**
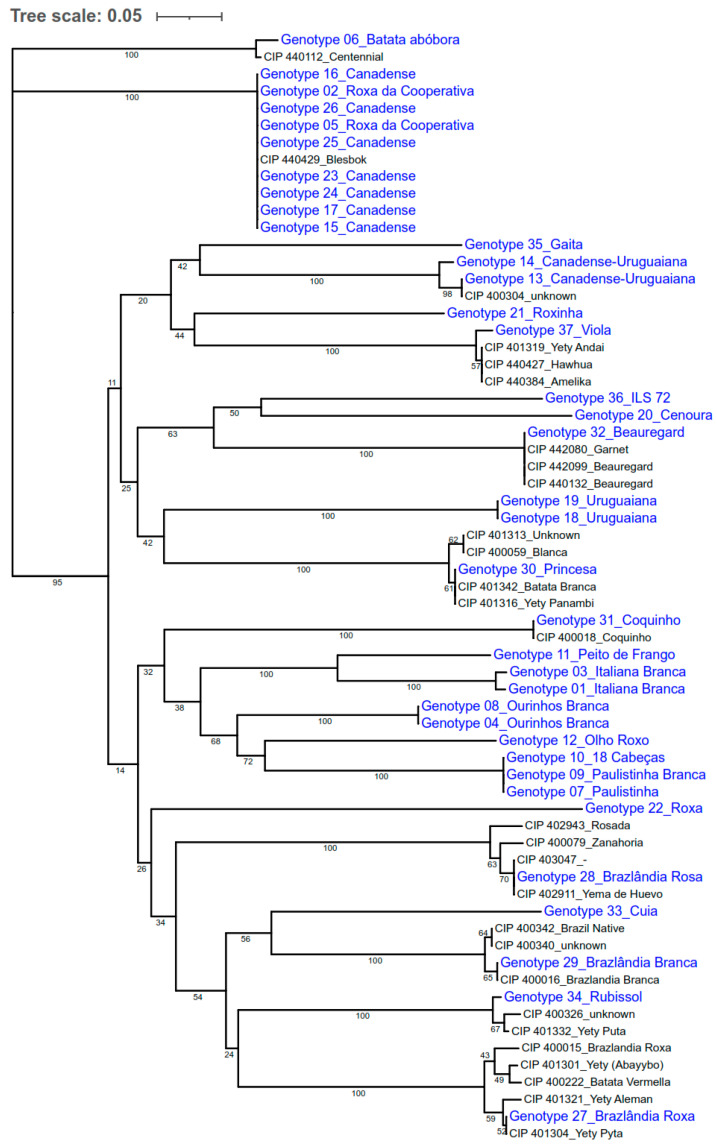
Neighbor joining dendrogram based on Jaccard distances, showing node-support percentages obtained from 1000 bootstrap resamplings, for the 37 samples collected in the current study (in blue) and 28 samples from International Potato Center Genebank.

The tree constructed using the genetic dissimilarity matrix among accessions, calculated with the Jaccard coefficient, showed that 20 of the 37 samples used in the current study matched with 28 accessions already present at CIP (Table 4), forming 11 different clusters. The resulting tree (Figure 4) aims to illustrate the similarity relationships among individuals, but it is not intended to represent phylogenetic relationships between accessions or clusters.

The tree revealed several groups of accessions with zero pairwise distance, indicating an absence of allelic variation among them (Figure 4). These cases correspond to genetically identical profiles and therefore represent duplicates within the analyzed set of samples. Additional groups showed minimal differentiation, with Jaccard distances below the established 0.05 threshold, suggesting that they may also constitute potential duplicates or clonal lineages derived from the same genotype. A ROC curve was elaborated to indicate specificity and sensitivity, further supporting results can be found in Supplementary Figure S1.

CIP has more than 100 sweetpotato genotypes from Brazil in its collection. The registered cultivar samples shipped from Embrapa clustered with accessions of the same genotypes priorly sent to CIP’s Genebank in 1991 (e.g., ‘Coquinho’, ‘Brazlândia Branca’, and ‘Brazlândia Roxa’). Nine accessions collected from commercial growers in different regions of Brazil (São Paulo, Brasília, and Sergipe) clustered with ‘Blesbok’, an improved variety originating from South Africa [24], introduced into the CIP collections as CIP 440429. Another sample, commonly planted in the region of Patrocínio, MG (Canadense-Uruguaiana), clustered with the accession CIP 400304, also from Brazil. Interestingly, most of the samples collected from Sergipe state did not match any accession from the CIP collection. At least five unique samples were observed, demonstrating that some genetic pools are still missing at CIP’s collection (Figure 4).

### 3.4. Morphological Evaluation

**Figure 5:**
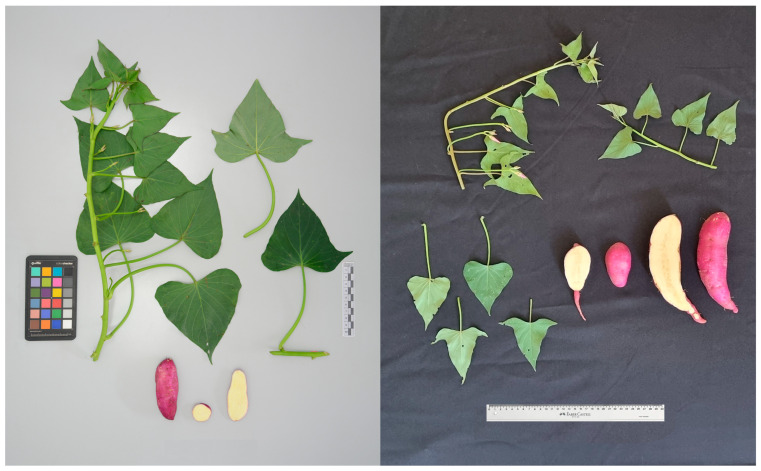
Similarity of foliage and storage root of sweetpotato accession Blesbok preserved at the International Potato Center (CIP) (**left**) and one representative of the genotypes collected in Brazil as part of this study that clustered with Blesbok based on the SSR analysis (**right**).

Some similarity was observed between the morphological results obtained from the field evaluations performed at Embrapa and the morphological descriptors in the CIP database for the accessions that clustered together. In the first cluster, CIP 440112 and Genotype 06 exhibited similar orange skin and flesh colors, but some foliar characteristics were slightly different, such as plant type, pubescence, and immature foliage color. The nine field samples collected in this study that molecularly clustered with the South African cultivar ‘Blesbok’ (CIP 440429) displayed the same morphology as the cultivar across all the morphological characteristics evaluated. This includes the same spreading plant type, green vine pigmentation, storage roots with purple-red skin, pale yellow flesh, and no secondary flesh color (Figure 5). Interestingly, both the plants evaluated in this study and those characterized at CIP exhibited similar variability in leaf shape, both within plants and within the same plant (Figure 5). Plants of this genotype can present hastate and cordate leaf shapes (Figure 5).

None of the genotypes from Sergipe, which have roots commercialized as ‘white skin sweetpotato’ in Brazilian supermarkets, clustered with any CIP accession. However, when carefully evaluated and compared to the Munsell Color Chart, it is evident that genotype 11 exhibited white skin, genotypes 1, 3, 4, 7, 8, and 9 had cream-colored skin, and genotype 12 had dark cream-colored skin. Internally, the root flesh colors were also distinct, with genotypes 1, 3, 4, 8, and 11 presenting pale-yellow flesh, while genotypes 7, 9, and 12 had cream-colored flesh. The foliage of the five sub-clusters within the Sergipe cluster also differed in some of the attributes evaluated (Table 5). Genotype 10 was not included in the evaluation, as it was lost during in vitro processing at the time of planting. Eleven sweetpotato cultivars were utilized in the current study. The cultivars ‘Brazlândia Roxa’, ‘Brazlândia Branca’, ‘Coquinho’, and ‘Beauregard’ were already present at the CIP Genebank. These accessions, evaluated in this study, clustered molecularly with the expected similar accessions from CIP, and also presented the same morphology as the CIP plants. Additional data is provided in Supplementary Table S1.

## 4. Discussion

The agriculture and livestock sectors are constantly developing new cultivars, animal breeds, and production systems to meet the growing consumer demand. In this context, consumers are increasingly seeking information regarding food origin, production methods, nutritional properties, and the traceability of these products until they reach the market [25].

As a center of diversity of sweetpotato, Brazil has many unknown varieties traditionally cultivated in different regions, often identified regionally by producers using local names. One common aspect is that most of these commercially grown sweetpotato genotypes are not registered as cultivars with the Brazilian Ministry of Agriculture, Livestock, and Food Supply [8].

The ease of propagation of this crop by cuttings facilitates the transfer of samples among producers. However, this practice, combined with some degree of disease accumulation through clonal multiplication, can lead to a mixing of genotypes, resulting in the misidentification of known cultivars being planted. This issue makes the traceability process difficult and directly impacts the commercialization of the crop in the markets and the legality of selling cuttings by producers and companies. Therefore, characterizing the prevalent genotypes being planted and identifying their genetic origin can support the registration of these varieties with MAPA and help formalize the sweetpotato commercial chain in Brazil. This information could also aid sweetpotato breeding programs by guiding crosses among different genotypes.

One of the major findings of the current study is the confirmation that the Brazilian genotype known as ‘Canadense’ is directly related to the South African cultivar ‘Blesbok’ [24]. This cultivar was released in 1991 and remains one of the most important white flesh genotypes commercialized in its country of origin [26]. In Brazil, ‘Blesbok’ became one of the most planted genotypes due to its adaptability to different regions [27,28] and resistance to pathogens [29].

The sole use of morphological descriptors can lead to improper identification of a variety since these descriptors can vary according to the environmental conditions and even within the same plant. Additionally, much information, such as nutrient content and pest and pathogen resistance, is usually not captured through these types of characterization. The identification of ‘Canadense’s origin was made possible by a strategy that combined both morphological and molecular evaluations. It is important to highlight that relying solely on morphological evaluation would have misled the comparison between the CIP accession and the nine samples in this study, as ‘Blesbok’ exhibits more than one type of mature and immature leaf shape (hastate and cordate), and the root shape is also variable within the same plant.

The exclusive use of the molecular approach also presents some limitations, particularly regarding the presence/absence scoring approach used for SSR data. In this method, each allele is treated as an independent marker, and the absence of allele dosage information limits the ability to capture true allelic proportions in this polyploid species. As a result, within-locus variability and partial heterozygosity may be underestimated under this binary scoring scheme. However, these limitations do not compromise the main objective of this study, which focuses on the discrimination and identification of individual genotypes rather than on the precise estimation of allelic diversity levels. Therefore, the inclusion of SSR information [10] in combination with the morphological characterization and the availability of a global molecular database were crucial in confirming the identity of the variety. The confirmation of the origin of this genotype is important for commercial growers in Brazil because it enables the registration and formalization of its use as a cultivar in the country.

Another relevant finding of this study is that the local varieties from Sergipe were not associated with any known cultivar or landrace maintained at CIP. This observation demonstrates that, despite hosting the largest and most diverse sweetpotato collection in the world, some genetic diversity is still missing at CIP’s collection. Additionally, this finding demonstrates the relevance of some Brazilian regions as potential sites of diversification of the crop. Therefore, new initiatives to increase the diversity of South America accessions at CIP’s collections should be considered to ensure the long-term preservation of this valuable diversity.

Given the importance of this type of study, similar initiatives have been recorded in the literature. In Tanzania, 136 sweetpotato landraces collected from three different agroecological zones were evaluated morphologically and agronomically. It was observed that many varieties, named differently across the three zones, showed close resemblance and were grouped into similar clusters, suggesting the presence of synonyms [30]. The same study also identified that several morphologically distinct landraces were named similarly to some improved cultivars by some local communities. The authors highlighted that this diverse system of naming cultivars not only limited the proper identification of cultivars but also hindered the monitoring and follow-up of newly released improved cultivars from research stations once they reach the farmers. Therefore, comprehensive information concerning locally available sweetpotato germplasm is of vital importance for the advancement of breeding work.

In another similar study, the use of morphological and agronomical characterization in different agricultural zones in Cameroon demonstrated high variation among the sweetpotato varieties evaluated [31]. However, the authors suggested that grouping sweetpotato varieties based exclusively on morphological characteristics can present some limitations [31].

Molecular data can significantly enhance the identification of dissimilarities among genotypes and the detection of potential duplicates. However, since sweetpotato is a hexaploid species, with the potential effect of allele dosage on gene expression, a thorough interpretation of molecular qualitative data is required. Previous studies using morphological traits, qualitative characteristics, and molecular markers to compare accessions from Italy and South America highlighted the relevance of a broad approach for genotype identification [32]. The authors found that combining various characterization methods allowed for the differentiation or clustering of sweetpotato genotypes according to their geographical origin and phenotypic descriptors. They emphasized that this information could be leveraged by breeders and farmers for the detection and protection of commercial varieties, thereby facilitating traceability efforts.

The findings from this study serve as a road map for other initiatives that aim to improve the traceability of variety commercialization. The approach utilized in this study directly enabled the identification of relevant genotypes. The association of virus elimination with genotyping, phenotyping, and comparison of this information with that available at an International Research Center will support the registration of these accessions with MAPA, thereby improving the formalization of the sweetpotato commercial chain in Brazil. Additionally, these findings can assist sweetpotato breeding programs in planning crosses among different genotypes.

Our findings show that discriminatory power is unevenly distributed across the 19 SSR loci and that cumulative resolution plateaus after the inclusion of 11 loci. This pattern indicates redundancy among markers and highlights that loci with higher allele richness and higher PIC or Nei’s diversity contribute differently to genotype differentiation. The subset of 11 loci (IBS28, IBS139, IBS11, IbJ522a, IbJ116A, IbY60, IBS30, IBC12, IBS141I, BS44, and IBS199) can uniquely identify accessions in the present dataset.

These results demonstrate that marker choice, more than the number of loci, determines the ability to separate genotypes at short genetic distances. This finding refines the design of SSR panels for sweetpotato curation and routine verification, emphasizing the selection of highly informative loci over increasing marker count.

## 5. Conclusions

This study demonstrates that integrating SSR molecular markers with detailed morphological assessment and the use of a broad database offers a robust framework for distinguishing sweetpotato genotypes, even in the context of mixed-slip propagation and cultivar misidentification. SSR markers provided high discrimination power, confirming their effectiveness for genotype differentiation. Crucially, the genotype locally known as “Canadense” consistently clustered with the CIP accession called ‘Blesbok’, which originated from a South African cultivar, both genetically and phenotypically, offering clear evidence to support its registration and formalization in Brazil’s seed and plant material system. Conversely, multiple accessions from Sergipe did not match any CIP reference, highlighting the presence of novel, regionally distinct genotypes.

## Figures and Tables

**Table 1:** Sweetpotato samples utilized in the current study.

Identification	Grower Site *	Variety Local Name	Biological Status
Genotype 1	Sergipe, SE	Italiana Branca	Variety
Genotype 2	Sergipe, SE	Roxa da Cooperativa	Variety
Genotype 3	Sergipe, SE	Italiana Branca	Variety
Genotype 4	Sergipe, SE	Ourinhos Branca	Variety
Genotype 5	Sergipe, SE	Roxa da Cooperativa	Variety
Genotype 6	Sergipe, SE	Batata abóbora	Variety
Genotype 7	Sergipe, SE	Paulistinha	Variety
Genotype 8	Sergipe, SE	Ourinhos Branca	Variety
Genotype 9	Sergipe, SE	Paulistinha Branca	Variety
Genotype 10	Sergipe, SE	18 Cabeças	Variety
Genotype 11	Sergipe, SE	Peito de Frango	Variety
Genotype 12	Sergipe, SE	Olho Roxo	Variety
Genotype 13	Minas Gerais, MG	Canadense-Uruguaiana	Variety
Genotype 14	Minas Gerais, MG	Canadense-Uruguaiana	Variety
Genotype 15	São Paulo, SP	Canadense	Variety
Genotype 16	São Paulo, SP	Canadense	Variety
Genotype 17	São Paulo, SP	Canadense	Variety
Genotype 18	São Paulo, SP	Uruguaiana	Variety
Genotype 19	São Paulo, SP	Uruguaiana	Variety
Genotype 20	São Paulo, SP	Cenoura	Variety
Genotype 21	São Paulo, SP	Roxinha	Variety
Genotype 22	São Paulo, SP	Roxa	Variety
Genotype 23	Brasília, DF	Unknown	Variety
Genotype 24	Brasília, DF	Unknown	Variety
Genotype 25	Brasília, DF	Unknown	Variety
Genotype 26	Brasília, DF	Unknown	Variety
Genotype 27	CNPH, DF	Brazlândia Roxa	Cultivar
Genotype 28	CNPH, DF	Brazlândia Rosada	Cultivar
Genotype 29	CNPH, DF	Brazlândia Branca	Cultivar
Genotype 30	CNPH, DF	Princesa	Cultivar
Genotype 31	CNPH, DF	Coquinho	Cultivar
Genotype 32	CNPH, DF	Beauregard	Cultivar
Genotype 33	ECT, RS	Cuia	Cultivar
Genotype 34	ECT, RS	Rubissol	Cultivar
Genotype 35	ECT, RS	Gaita	Cultivar
Genotype 36	ECT, RS	ILS 72	Cultivar
Genotype 37	ECT, RS	Viola	Cultivar

* CNPH. Embrapa Vegetables, Brasília, DF, ECT. Embrapa Temperate Agriculture, Pelotas, RS.

**Table 2:** Marker statistics and diversity estimates, including total number of alleles, allele size, mean number of alleles per individual, Nei’s gene diversity, and Polymorphism Information Content (PIC) value from sweetpotato samples obtained in this study and samples from the International Potato Center (CIP). The absolute differences between corrected and uncorrected values were divided by the uncorrected values to calculate the percentage of change.

SSR Marker	Allele Sizes (bp)	Total Number of Alleles	Mean Allele Number			Nei’s Gene Diversity ^1^			PIC ^2^		
			Total	CIP	Current Study	Without Correction	Corrected by Sample Size	% Change *	Without Correction	Corrected Allele Frequencies	% Change *
IBS11	236–260	8	3.42	3.21	3.58	0.817	0.820	0.46	0.79	0.81	2.28
IBS141	123–149	11	4.38	4.36	4.41	0.870	0.873	0.35	0.86	0.84	1.83
IBS199	171–212	12	4.09	4.32	3.92	0.857	0.860	0.38	0.84	0.82	2.79
IbJ116A	205–250	10	3.45	3.21	3.62	0.801	0.804	0.44	0.77	0.71	8.18
IbE2	109–142	11	3.22	2.89	3.46	0.836	0.840	0.43	0.82	0.81	1.22
IBS30	189–241	9	3.48	3.39	3.54	0.787	0.791	0.46	0.76	0.72	4.96
IBS82	141–163	8	3.68	4.09	3.43	0.814	0.818	0.45	0.79	0.78	1.13
IBS28	187–228	11	4.42	4.21	4.58	0.852	0.855	0.36	0.84	0.82	2.19
IBC12	111–132	8	3.65	3.68	3.62	0.813	0.817	0.42	0.79	0.76	3.71
IBS139	304–348	12	3.46	3.29	3.59	0.838	0.842	0.44	0.82	0.82	0.60
IBY44	183–216	8	4.06	4.07	4.05	0.829	0.832	0.37	0.81	0.79	1.58
IBS44	127–151	8	3.46	3.39	3.51	0.844	0.848	0.45	0.83	0.83	0.02
IbJ10a	191–220	7	3.41	3.30	3.50	0.841	0.845	0.46	0.82	0.84	1.74
IbJ522a	226–265	5	2.26	2.25	2.27	0.664	0.668	0.53	0.62	0.56	9.11
IBS14	192–200	3	2.08	2.11	2.05	0.642	0.645	0.52	0.57	0.64	13.37
IbJ1809	143–155	5	3.36	3.57	3.19	0.778	0.782	0.47	0.74	0.73	1.42
IBY60	188–206	8	3.76	4.15	3.47	0.812	0.816	0.43	0.79	0.77	2.04
IBS24	149–169	6	3.53	3.56	3.51	0.781	0.785	0.44	0.75	0.72	4.25
IbJ544b	191–211	6	2.17	2.04	2.27	0.625	0.629	0.58	0.56	0.55	3.28

^1^ Nei’s gene diversity corrected by sample size [17]. ^2^ PIC calculated with corrected allele frequencies [15]. * Percentage of variation after correction was obtained by calculating the absolute differences between corrected and uncorrected values and dividing by the initial uncorrected value.

**Table 3:** Polymorphism, number of banding patterns, and Discriminating power (D) for the nineteen SSR loci.

SSR Marker	NP	I	C	D	DL
IBS11	8	21	0.07	0.93	0.91
IBS141	11	21	0.06	0.94	0.92
IBS199	12	21	0.06	0.94	0.92
IbJ116A	10	21	0.08	0.92	0.91
IbE2	11	20	0.07	0.93	0.92
IBS30	9	16	0.10	0.90	0.89
IBS82	8	19	0.11	0.89	0.88
IBS28	11	22	0.06	0.94	0.93
IBC12	8	18	0.10	0.90	0.89
IBS139	13	22	0.06	0.94	0.92
IBY44	8	16	0.09	0.91	0.90
IBS44	8	21	0.06	0.94	0.92
IbJ10a	7	19	0.07	0.93	0.91
IbJ522a	5	8	0.15	0.85	0.84
IBS14	3	6	0.21	0.79	0.78
IbJ1809	5	13	0.13	0.87	0.86
IbY60	8	20	0.10	0.90	0.89
IBS24	6	11	0.14	0.86	0.85
IbJ544b	6	8	0.27	0.73	0.72

NP = Number of polymorphic bands; I = (Average) number of patterns/assay unit; C = Average confusion probability; D = Average discriminating power; DL = Average limit of discriminating power.

**Table 4:** Sweetpotato landraces and improved varieties (ImpVariety) hosted at the International Potato Center (Lima, Peru) clustered with samples evaluated in the current study.

Accession Number	Country of Origin	Biological Status	Digital Object Identifier (DOI)	Accession Name
CIP400015	Brazil	ImpVariety	10.18730/2C8=	Brazlandia Roxa
CIP400016	Brazil	ImpVariety	10.18730/2C9U	Brazlandia Branca
CIP400018	Brazil	ImpVariety	10.18730/2CB1	Coquinho
CIP400059	Argentina	LandRace	10.18730/2DC$	Blanca
CIP400079	Argentina	LandRace	10.18730/2DWD	Zanahoria
CIP400222	Brazil	LandRace	10.18730/2J0$	Batata Vermella
CIP400304	Brazil	Landrace	10.18730/2MD0	Unknown
CIP400326	Brazil	Landrace	10.18730/2N2N	Unknown
CIP400340	Brazil	Landrace	10.18730/2NF$	Unknown
CIP400342	Brazil	LandRace	10.18730/2NG=	Brazil Native
CIP401301	Paraguay	LandRace	10.18730/3AK7	Yety (Abayybo)
CIP401304	Paraguay	LandRace	10.18730/3APA	Yety Pyta
CIP401313	Paraguay	LandRace	10.18730/3AZK	Unknown
CIP401316	Paraguay	LandRace	10.18730/3B2P	Yety Panambi
CIP401319	Paraguay	LandRace	10.18730/3B5S	Yety Andai
CIP401321	Paraguay	LandRace	10.18730/3B7V	Yety Aleman
CIP401332	Paraguay	LandRace	10.18730/3BJ1	Yety Puta
CIP401342	Paraguay	LandRace	10.18730/3BWB	Batata Branca
CIP402911	Argentina	LandRace	10.18730/3KEZ	Yema de Huevo
CIP402943	Argentina	LandRace	10.18730/3KR4	Rosada
CIP403047	Argentina	LandRace	10.18730/P64NW	Unknown
CIP440112	United States	ImpVariety	10.18730/654J	Centennial
CIP440132	United States	ImpVariety	10.18730/65R1	Beauregard
CIP440384	Tonga	LandRace	10.18730/6D8K	Amelika
CIP440427	Philippines	LandRace	10.18730/6EGP	Hawhua
CIP440429	South Africa	ImpVariety	10.18730/P79T~	Blesbok
CIP442080	United States	ImpVariety	10.18730/7FC1	Garnet
CIP442099	United States	ImpVariety	10.18730/19JEJ9	Beauregard

**Table 5:** Major foliage differences observed on the accessions collected on commercial fields in Sergipe state, Brazil.

Genotypes	Plant Type	Vine Length	Vine 1º Pigmentation	Vine Pubescens	General Outline	Foliage Color-Immature Leaf Color
Genotype 11	Erect	Very short	Green	Sparse	Lobed	Green
Genotype 01	Erect	Very short	Green	Absent	Triangular	Green
Genotype 03						
Genotype 04	Semi-erect	Short	Mostly dark purple	Moderate	Lobed	Green
Genotype 08						
Genotype 12	Semi-erect	Short	Green	Moderate	Hastate	Green with purple edge
Genotype 07	Erect	Short	Green	Absent	Lobed	Green with purple edge
Genotype 09						

## Data Availability

The datasets generated and analyzed during the current study are available from the corresponding author upon reasonable request.

## Supplementary Materials

The following supporting information can be downloaded at: https://www.mdpi.com/article/10.3390/biology15010091/s1. Figure S1: ROC curve of Jaccard distances as a predictor of duplicate groups. Blue points and their values in the graphic correspond to different Jaccard threshold distances. Table S1: Foliage and root descriptors* from sweetpotato samples obtained in this study.
