# Supplementary material for: Deciphering the Origins of Commercial Sweetpotato Genotypes Using International Genebank Data
Source: Biology (Basel). 2026 Jan 1;15(1):91. doi: 10.3390/biology15010091 (PMC12784737; doi:10.3390/biology15010091)
Supplement: Supplementary file 1 [file biology-15-00091-s001.zip › biology-3910634-supplementary materials.pdf]

Annex

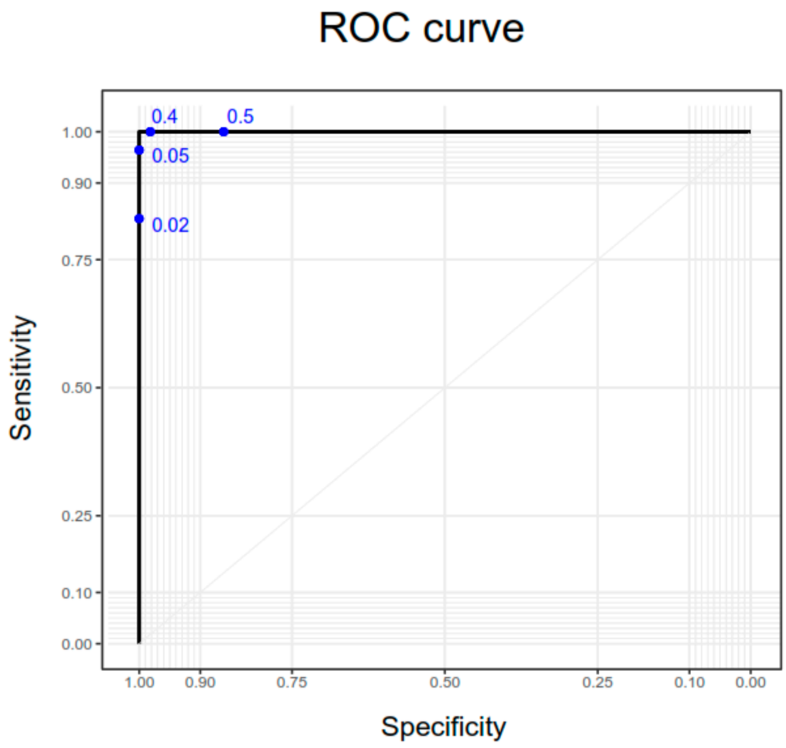

Threshold	Sensitivity	Specificity
0.007	0.634	1.000
0.015	0.652	1.000
0.015	0.661	1.000
0.015	0.670	1.000
0.016	0.705	1.000
0.016	0.723	1.000
0.016	0.741	1.000
0.017	0.777	1.000
0.017	0.795	1.000
0.019	0.821	1.000
0.025	0.830	1.000
0.029	0.848	1.000
0.030	0.866	1.000
0.031	0.875	1.000
0.031	0.884	1.000
0.033	0.893	1.000
0.039	0.911	1.000
0.044	0.929	1.000
0.046	0.938	1.000
0.048	0.955	1.000
0.050	0.964	1.000
0.062	0.982	1.000
0.071	0.991	1.000
0.161	1.000	1.000
0.248	1.000	0.999
0.298	1.000	0.999
0.346	1.000	0.996
0.356	1.000	0.995
0.364	1.000	0.993
0.369	1.000	0.988

Fig S1. ROC curve of Jaccard distances as predictor of duplicate groups. Blue points and their values in the graphic correspond to different Jaccard threshold distances.

Supplemental Table S1. Foliage and root descriptors* from sweetpotato samples obtained on this study.

Genotypes	Plant Type	Vine internode diameter	Vine internode length	Vine predominant pigmentation	Vine secondary pigmentation	Vine tip pubescence	General outline of the leaf	Leaf lobe type
G-01	3	4	1	1	0	0	4	0
G-02	7	3	3	1	0	3	6	3
G-03	3	4	1	1	0	0	4	0
G-04	5	3	3	7	0	5	6	5
G-05	7	3	3	1	0	3	6	3
G-06	3	3	1	1	0	0	6	3
G-07	3	1	3	1	0	0	6	3
G-08	5	3	3	7	0	5	6	5
G-09	3	1	3	1	0	0	6	3
G-11	3	3	1	1	0	3	6	5
G-12	5	3	3	1	0	5	5	5
G-13	7	1	5	7	0	0	6	7
G-14	7	1	5	7	0	0	6	7
G-15	7	3	3	1	0	3	6	3
G-16	7	3	3	1	0	3	6	3
G-17	7	3	3	1	0	3	6	3
G-18	7	3	3	1	6	3	4	0
G-19	5	3	3	1	6	3	4	0
G-20	3	3	1	1	0	0	4	0
G-21	3	3	1	7	1	0	6	5
G-22	5	3	3	5	0	0	4	0
G-23	7	3	3	1	0	3	6	3
G-24	7	3	3	1	0	3	6	3
G-25	7	3	3	1	0	3	6	3
G-26	7	3	3	1	0	3	6	3
G-27	5	4	3	1	0	3	4	0
G-28	7	4	5	1	0	3	6	5
G-29	3	5	1	1	0	5	4	0
G-30	7	3	4	7	1	0	5	7
G-31	3	1	1	1	3	0	6	5
G-32	7	3	4	1	0	0	4	0
G-33	3	3	1	1	0	0	6	3
G-34	7	4	3	1	0	5	6	3
G-35	7	3	4	1	0	3	4	0
G-36	5	3	1	1	0	3	4	0
G-37	5	3	1	1	0	0	6	5

Genotypes	Leaf lobe number	Shape of central lobe	Mature leaf size	Abaxial leaf vein pigmentation	Mature leaf color	Immature leaf color	Petiole pigment ation	Storage root shape
G-01	0	0	5	3	2	2	1	9
G-02	3	4	5	2	2	3	1	5
G-03	0	0	5	3	2	2	1	9

G-04	5	4	5	5	2	2	5	9
G-05	3	4	5	2	2	3	1	5
G-06	3	4	5	2	2	6	2	8
G-07	3	4	5	2	2	3	1	9
G-08	5	4	5	5	2	2	5	9
G-09	3	4	5	2	2	3	1	9
G-11	3	4	5	2	2	2	1	9
G-12	5	5	5	2	2	3	1	9
G-13	5	5	5	8	2	7	9	9
G-14	5	5	5	8	2	7	9	9
G-15	3	4	5	2	2	3	1	5
G-16	3	4	5	2	2	3	1	5
G-17	3	4	5	2	2	3	1	5
G-18	0	0	3	3	2	3	2	1
G-19	0	0	3	3	2	3	2	1
G-20	0	0	5	2	2	2	3	9
G-21	5	6	3	6	2	3	9	9
G-22	0	0	5	2	2	3	1	9
G-23	3	4	5	2	2	3	1	5
G-24	3	4	5	2	2	3	1	5
G-25	3	4	5	2	2	3	1	5
G-26	3	4	5	2	2	3	1	5
G-27	0	0	5	2	2	2	1	9
G-28	3	4	5	3	2	2	1	9
G-29	0	0	5	7	2	2	3	9
G-30	5	5	5	6	2	6	2	9
G-31	3	2	5	7	2	2	3	5
G-32	0	0	5	3	2	6	2	3
G-33	3	4	5	6	2	3	8	4
G-34	5	2	5	2	2	3	1	9
G-35	0	0	5	2	2	3	1	9
G-36	0	0	3	5	2	2	3	6
G-37	5	6	5	2	2	3	1	9

Genotypes	Storage root surface defects	Storage root cortex thickness	Predominant skin color	Secondary skin color	Predominant flesh color	Secondary flesh color	Distribution of secondary flesh color
G-01	0	7	2	0	4	0	0
G-02	0	5	8	0	4	0	0
G-03	0	7	2	0	4	0	0
G-04	0	5	2	0	4	0	0
G-05	0	5	8	0	4	0	0
G-06	0	7	4	0	7	0	0
G-07	0	5	2	0	2	0	0
G-08	0	5	2	0	4	0	0

G-09	0	5	2	0	2	0	0
G-11	0	7	2	0	4	0	0
G-12	0	7	2	0	3	0	0
G-13	5	7	8	0	2	0	4
G-14	5	7	8	0	2	0	4
G-15	0	5	8	0	4	0	0
G-16	0	5	8	0	4	0	0
G-17	0	5	8	0	4	0	0
G-18	0	3	8	8	2	0	0
G-19	0	3	8	8	2	0	0
G-20	0	3	5	0	7	0	0
G-21	0	7	8	0	2	0	0
G-22	0	5	8	8	9	0	0
G-23	0	5	8	0	4	0	0
G-24	0	5	8	0	4	0	0
G-25	0	5	8	0	4	0	0
G-26	0	5	8	0	4	0	0
G-27	0	7	8	0	4	0	0
G-28	0	7	6	0	4	0	0
G-29	0	9	2	0	4	0	0
G-30	0	7	3	0	4	0	0
G-31	0	1	2	0	2	0	0
G-32	0	5	5	0	6	0	0
G-33	0	3	2	0	2	0	0
G-34	0	7	8	0	2	0	0
G-35	0	5	3	0	4	0	0
G-36	5	5	5	0	8	0	0
G-37	0	3	8	0	2	0	0

*Foliage and root characterizations were performed 90 and 191 days after planting, respectively using descriptors previously reported [22].
